# Demographic and other correlates of non-prescription drug use among college students during the COVID-19 pandemic

**DOI:** 10.3389/fpubh.2025.1695969

**Published:** 2026-02-04

**Authors:** Subi Gandhi, Sidketa Fofana, Md Rafiul Islam, Tamer Oraby

**Affiliations:** 1 Department of Medical Lab Sciences, Public Health, and Nutrition Science, Tarleton State University, Stephenville, TX, United States; 2 School of Mathematical and Statistical Sciences, The University of Texas Rio Grande Valley, Edinburg, TX, United States; 3 Department of Mathematics and Statistics, University of the Incarnate Word, San Antonio, TX, United States

**Keywords:** college health, substance-related disorders, drug use, college students, COVID-19, pandemic

## Abstract

**Background and objectives:**

Substance use among college students in the U.S. remains a pressing concern and may have intensified during the COVID-19 pandemic due to increased stress, uncertainty, and academic disruptions. This study investigates the relationship between non-prescription drug use and various demographic, mental health, and behavioral factors among college students during the pandemic's early stages.

**Methods:**

Data were collected through online and in-person surveys in the summer semester of 2021. Behavioral health was assessed using validated instruments: the Patient Health Questionnaire-9 (PHQ-9) for depression and the Drug Abuse Screening Test-20 (DAST-20) for substance use. Demographic and behavioral variables were gathered via structured surveys through convenience sampling. Bivariate analyses were followed by logistic regression to identify significant predictors of drug use.

**Results:**

Preliminary analysis (*n* = 576) showed that 71.53% of participants were female, but non-prescription drug use was more commonly reported among male students (20.81%) than female students (13.11%). African American students reported the highest rate of non-prescription drug use (38.16%) among the different races/ethnicities explored for drug use behavior. Most participants were undergraduates (71.48%), and 44.10% identified as first-generation college students. Logistic regression (*n* = 503) revealed that African American students had nearly three times the odds of drug use compared to Caucasian students (OR = 2.962). Students with depressive symptoms were twice as likely to report drug use (OR = 2.023). Alcohol use was a strong predictor: students drinking two to four times monthly had over three times the odds of drug use compared to non-drinkers (OR = 3.223). Students aged 22–23 had nearly four times the odds compared to those aged 20 (OR = 3.976). However, gender, COVID-19 positivity status, first-generation status, academic classification, and on-campus residence were not significantly associated with drug use (*p* > 0.05).

**Conclusion:**

This study identifies key demographic and behavioral health correlates of non-prescription drug use during a time of heightened stress. The findings underscore the need for targeted behavioral health interventions in college settings, particularly during public health crises. Understanding these risk factors can inform campus health strategies and support services aimed at reducing substance use and promoting student wellbeing.

## 1 Introduction

### 1.1 History of substance use pre- and post-pandemic

Substance use has emerged as one of the most pressing behavioral health concerns on college campuses across the United States in recent years (1–5). While historically some forms of substance use were perceived as socially normative or even protective in certain contexts (2–4), contemporary research underscores its detrimental impact on the health, academic performance, and social wellbeing of students, as well as broader societal consequences (6, 7).

Even before the COVID-19 pandemic, college students exhibited elevated rates of substance use and mental health challenges. For example, data from 2015 to 2019 revealed that individuals aged 18–22 enrolled in college were significantly more likely than their non-college peers to misuse prescription stimulants, consume alcohol, and engage in binge drinking (5, 8).

The onset of the COVID-19 pandemic marked a pivotal shift in substance use patterns. During the early stages of the pandemic, alcohol consumption increased across the general population, while marijuana use displayed more heterogeneous trends (9–12). These behavioral shifts could be primarily attributed to pandemic-related stressors, including social isolation, economic uncertainty, and disruptions to daily routines. Stress relief and coping with loneliness emerged as primary motivations for increased alcohol use (13). Notably, younger individuals, males, and those experiencing economic hardship, such as job loss, were disproportionately affected (13).

Although substance use among college students during the pandemic remains relatively underexplored (14), available data reveal troubling trends. The 2022 Monitoring the Future study reported that 40.9% of college students aged 19–22 used cannabis in the past year, 26.4% vaped nicotine, 27.7% engaged in binge drinking, and 5.2% participated in high-intensity drinking within the past 2 weeks. A separate 2021 study estimated that 21.8% of college students met the criteria for a past-year substance use disorder (4). Furthermore, in 2022, alcohol use and binge drinking remained more prevalent among college students (80.5 and 27.7%, respectively) than among their non-college peers (72.7 and 23.9%) (15). These findings are supported by the 2023 National Survey on Drug Use and Health (NSDUH), which reported that 49.6% of full-time college students aged 18–25 consumed alcohol in the past month, with 29.3% engaging in binge drinking and 6.8% in heavy drinking (16, 17). Complementary national data show that nearly half of current student drinkers reported being drunk in the past 30 days, and 1 in 10 college students consumed more than 10 drinks in a row within the past 2 weeks (1, 18, 19).

### 1.2 Risk factors

The collegiate environment presents a unique constellation of stressors that contribute to the heightened prevalence of substance use among students. Academic pressures, social transitions, and the pursuit of independence often converge to create a high-risk context for maladaptive coping behaviors (5). In response to these challenges, many students may turn to substances such as alcohol, marijuana, and other drugs as mechanisms for stress relief and emotional regulation (5).

Beyond academic stress, interpersonal and developmental factors also play a critical role. Students may use substances to cope with unresolved family conflicts, peer relationship difficulties, or adverse childhood experiences (20, 21). The social fabric of college life, particularly the influence of peer norms and the pervasive “party culture,” further amplifies these risks. Membership in Greek organizations, such as fraternities and sororities, has been consistently linked to elevated rates of alcohol and marijuana use (5, 22). For individuals already vulnerable to substance misuse, participation in these groups can exacerbate risky behaviors and compound adverse health outcomes (22–24).

Psychological traits also contribute significantly to substance use vulnerability. Traits such as impulsivity, low self-esteem, and heightened anxiety have been identified as predictors of increased substance use among college students (25). One theoretical framework that elucidates this relationship is *self-control theory*, which posits that individuals with lower levels of self-regulation are more likely to engage in risky behaviors. Empirical studies have demonstrated that low self-control is associated with higher rates of marijuana use and prescription drug misuse in university populations. Moreover, this relationship is moderated by contextual variables such as peer influence and access to substances, underscoring the dynamic interplay between individual predispositions and environmental factors (26).

Additionally, the concepts of negative and positive urgency, the tendency to act impulsively in response to extreme emotional states, have been identified as key risk factors for escalating alcohol consumption during college. These urgency traits increase the likelihood of drinking to enhance positive emotions or to mitigate negative ones. Recent findings suggest that baseline alcohol use can predict an increase in enhancement motives for drinking, mediated by heightened positive urgency (27).

### 1.3 Consequences of substance use among college students

Substance use is associated with significant general medical, psychiatric, and non-psychiatric morbidity and mortality for many students (5, 21, 28). Although alcohol and opioids continue to be focal points of discussion, increasing marijuana usage and the misuse of prescription stimulants and other substances introduce additional layers to the issue.

#### 1.3.1 Alcohol and opioids

Alcohol and opioid use are common on college campuses. Between 1999 and 2008, hospitalization rates for alcohol overdoses among young adults aged 18–24 increased by 25%, drug overdoses by 55%, and combined alcohol and drug overdoses by 76%. Opioid-related overdoses surged by 122%, with alcohol involved in approximately 20% of these cases. Men were more likely than women to experience such co-occurrences (28). Alcohol overdoses occur when high levels of alcohol depress vital brain functions, including breathing, heart rate, and temperature regulation. Symptoms may include confusion, unconsciousness, vomiting, seizures, and respiratory depression, potentially resulting in brain damage or death (29). Similarly, opioid overdoses suppress the brain's respiratory centers, which can also lead to fatal outcomes when taken in excessive amounts (30).

Students who engage in heavy drinking or drug use often face academic challenges, such as reduced GPA, more frequent absences, less time spent studying, decreased motivation, and a higher likelihood of dropping out or experiencing unemployment after graduation (5, 31). The misconception that using stimulants enhances academic focus is prevalent, but the long-term effects are harmful (32). Substance use contributes to social and behavioral risks, including poor decision-making, interpersonal conflicts, and elevated rates of campus violence and sexual assault (5, 33).

#### 1.3.2 Marijuana and other common substances

Marijuana use among college students is increasing. In 2020, 44 percent reported past-year use, the highest in 35 years (34). Although often seen as less harmful, marijuana use has serious academic and cognitive consequences. Regular use impairs memory, attention, and learning, resulting in a lower GPA, increased procrastination, class absences, and delays in graduation (35, 36). Additionally, college students commonly misuse stimulants (e.g., Adderall and Ritalin), hallucinogens (e.g., LSD and psilocybin), and sedatives (e.g., Xanax and Valium), often seeking academic enhancement, recreation, or stress relief. However, nonmedical use of these substances can lead to serious health risks, including addiction, psychological distress, impaired cognition, and potentially fatal overdoses, especially when mixed with other drugs or alcohol (37–39).

#### 1.3.3 Co-occurrence of mental health and substance use

Substance use and mental health issues often do not occur in isolation; instead, they frequently co-occur and can intensify one another, particularly among young adults (40). Research consistently shows that students experiencing higher levels of depression or anxiety are also more likely to engage in frequent substance use, indicating a strong behavioral and psychological overlap (41). This pattern has been observed over the past two decades and is often associated with ineffective coping strategies commonly employed by students (42). Many individuals with depressive symptoms turn to substances as a form of self-medication in an attempt to alleviate emotional distress (43, 44). However, the pharmacological effects of substances, such as alcohol, a central nervous system depressant, and other drugs that disrupt mood or sleep, can trigger or worsen symptoms of depression and anxiety. The challenges of substance use and mental health disorders profoundly affect individuals, families, communities, and society as a whole (45).

### 1.4 Study rationale

Although substance use among college students has been widely studied, there remains a significant gap in understanding how pandemic-related stressors, such as isolation, academic disruption, and uncertainty, shaped substance use behaviors during the early phases of the COVID-19 pandemic, a period marked by heightened psychological vulnerability (46). In this study, we examined the following research questions:

1. Which demographic variables (e.g., age, gender, race/ethnicity) were associated with substance use during the study period?
2. What was the prevalence of substance use and abuse among college students during the study period?
3. Is there a correlation between mental health outcomes (e.g., depression, anxiety) and substance use behaviors?

## 2 Materials and methods

### 2.1 Study design and setting

This study employed a cross-sectional design and was conducted at a large public university in Central Texas during the COVID-19 pandemic. Data collection occurred between June 25 and July 16, 2021, capturing student experiences during a period of heightened psychological stress and academic disruption.

### 2.2 Participants

Participants included undergraduate and graduate students recruited through convenience sampling. Recruitment was facilitated via university-wide email invitations containing a secure Qualtrics survey link and printed surveys distributed in high-traffic campus locations such as the dining hall, library, and student center. Eligibility required current enrollment and completion of survey items related to substance use and mental health.

### 2.3 Variables of interest

The structured survey comprised multiple sections designed to capture a broad range of variables related to student wellbeing and behaviors during the COVID-19 pandemic. The development of the instrument was informed by previously validated scales and peer-reviewed literature, with particular emphasis on mental health and substance use domains. These areas were selected based on emerging research trends during the pandemic, ensuring relevance and rigor in the survey design (47–56).

1. Demographic information: age, gender identity, race/ethnicity, degree level (undergraduate, graduate, postgraduate, other), first-generation college student status, and current campus residency.
2. Mental health: the Patient Health Questionnaire-9 (PHQ-9) was used to assess depressive symptoms. The Generalized Anxiety Disorder-7 (GAD-7) scale assessed anxiety symptoms. Both measures have demonstrated high reliability in similar populations (47, 57). Due to multicollinearity between depression and anxiety scores, an issue frequently encountered in mental health research owing to symptom overlap (58, 59), we designated the PHQ-9 depression score as the primary outcome variable for analysis. This choice was made to mitigate redundancy and enhance model stability, as high correlations between PHQ-9 and GAD-7 scores can pose challenges to statistical interpretation (58, 59).
3. Substance use: the Drug Abuse Screening Test (DAST-20) is a widely utilized instrument for identifying potential drug abuse issues across diverse populations (60, 61). Comprising 20 straightforward yes/no questions, the DAST-20 has demonstrated acceptable reliability and validity in global applications (61). In our study, we initially collected responses from 571 participants. However, 68 were excluded due to incomplete answers on the DAST-20, resulting in a final analytic sample of 503 respondents.
4. COVID-19 experiences: students reported their COVID-19 test history (positive or negative), perceived risk of infection, and pandemic-related disruptions (e.g., isolation, academic stress).
5. Measures of additional pandemic-related stressors are reported elsewhere (47, 54, 55, 62). These variables were not included in the current study, as they did not align with the specific association we aimed to investigate.
6. This self-administered survey approach provided a flexible and low-risk method for data collection, aligning with institutional policies and ethical guidelines during an active public health emergency. The mixed-method data collection (online and printed) facilitated greater reach, particularly for students who are less engaged with university email systems.

### 2.4 Data sources and measurement

Data were collected using a structured survey informed by validated instruments and peer-reviewed literature. Data collection took place between June 25 and July 16, 2021, during the COVID-19 pandemic. Participants were recruited through two primary methods: university-wide email invitations containing a study overview and a secure Qualtrics survey link, and the distribution of printed surveys in high-traffic campus locations such as the dining hall, library, and student center.

Age was categorized into discrete groups (e.g., 18, 19, 20, 21, 22–23, 24–31, 32+) to reflect varying levels of exposure to high-risk behaviors across the college experience. This approach allowed us to examine how students' engagement with behaviors such as alcohol and non-prescription drug use may differ depending on their age and college tenure. For example, 18-year-old freshmen are typically new to the college environment and may have limited exposure to high-risk social settings compared to older students. In contrast, students aged 21 and older—who have reached the legal drinking age—may exhibit different patterns of substance use due to increased access and social acceptance. Data from SAMHSA show that the number of college students meeting criteria for alcohol use disorder more than doubles from age 18 (104,000) to age 21 (231,000) (63). Additionally, binge drinking and heavy alcohol use are significantly more prevalent among full-time college students aged 21 and older. By structuring age into these specific categories, we aim to capture meaningful differences in behavioral risk profiles that align with legal thresholds and experiential factors (63).

The frequency of alcohol use is directly linked to health risks such as liver disease, mental health disorders, and alcohol use disorder (AUD) (64). Studies show that even moderate drinking can increase health risks, and higher frequency correlates with greater harm. Alcohol use was categorized by frequency (e.g., never, monthly, 2–4 times/month).

In alignment with established recommendations for depression screening in general medical populations, we used a PHQ-9 cut-off score of 10 to classify depression as a binary variable (presence vs. absence). This threshold has been widely validated and is associated with optimal sensitivity (88%) and specificity (85%) for detecting major depressive disorder across diverse settings, making it a reliable choice for screening in non-psychiatric populations (65–67).

For the assessment of drug use, we utilized the first item from the Drug Abuse Screening Test (DAST-20): “*Have you used drugs other than those required for medical reasons?*” This item served as a binary indicator of non-medical drug use (yes/no). This enabled us to capture self-reported illicit drug use, retaining statistical power and aligning with screening protocols. DAST-20 is valued for its simplicity, reliability, and effectiveness in clinical and research settings to evaluate substance use (60).

### 2.5 Bias

To address potential bias from missing data, multiple imputation was performed using the Amelia package in R (68). This method applies an expectation-maximization algorithm with bootstrapping to generate plausible values for incomplete observations, preserving dataset integrity and reducing bias associated with listwise deletion.

### 2.6 Study size

A total of 576 students participated in the study. The final analytic sample for logistic regression included 503 students who provided complete responses to the DAST-20 items.

### 2.7 Statistical software and methods

Traditional analytical methods, machine learning procedures, and data visualization were conducted using a combination of statistical software packages, including R, Python, and SAS. To address missing data, we employed multiple imputations using the *Amelia* package in R (68), which generates plausible values for incomplete observations based on an expectation-maximization algorithm with bootstrapping (69). This method was selected to preserve the integrity of the dataset and reduce potential biases associated with listwise deletion or other *ad hoc* imputation techniques. Amelia provides powerful methods for fast, robust multiple imputations without sacrificing the statistical rigor of uncertainty quantification (69).

Descriptive statistics were used to summarize demographic and behavioral characteristics. Bivariate associations between drug use and categorical variables were examined using Chi-square tests. Logistic regression was conducted to identify independent predictors of drug use, with odds ratios and 95% confidence intervals reported. A Random Forest classifier was applied to imputed DAST-20 data to enhance predictive modeling. To address class imbalance, the Synthetic Minority Oversampling Technique (SMOTE) was used. Model performance was evaluated using accuracy, precision, recall, and F1 score, and feature importance scores were calculated to assess the contribution of each predictor.

## 3 Results

### 3.1 Participants and descriptive data

Supplementary Table S1 presents the results of bivariate analyses examining the associations between drug use and various demographic, behavioral, and other factors (*n* = 576). Chi-square tests were used to assess independence between drug use status and categorical variables, including age group, race/ethnicity, gender, alcohol use, COVID-19 test result, first-generation college status, degree level, campus residency, and depression status. Among the 576 participants, 89 (15.45%) reported using non-prescription drugs. Drug use prevalence was higher among men (20.81%) than women (13.11%). The highest rate of drug use was observed among students aged 22–23 years (25.93%). African American students reported the highest prevalence of drug use (38.16%). Most respondents were female (71.53%), undergraduate (71.48%), and nearly half (44.10%) identified as first-generation college students. Only 31.65% live on campus. Statistically significant associations were found between drug use and age, race/ethnicity, gender, alcohol use, and depression status (*p* < 0.05), while no significant associations were found for COVID-19 positivity, first-generation status, degree level, or campus residency.

### 3.2 Outcome data

Among students who drank alcohol two to four times per month, 50% reported drug use, compared to 12.04% among non-drinkers. Of the 503 students included in the logistic regression analysis (Table 1), 348 received a DAST-20 score of 0, while 155 scored 1 or higher. Depression was reported by 38.39% of participants. Among those with depressive symptoms, 24.11% reported drug use, compared to 10.67% among those without symptoms.

**Table 1:** Logistic regression analysis of factors associated with DAST-20 drug abuse (yes/no) (*n* = 503).

**Variable**	**df**	**Estimate (β)**	**Std error**	***p*-value**	**Odds ratio**	**Odds ratio (95% CI)**
Intercept	1	0.3868	0.4485	0.3885		
**Gender (ref: male)**						
Female	1	−0.3284	0.2190	0.1338	0.814	0.505–1.312
Others	1	0.4513	0.3905	0.2478	1.776	0.537–5.875
**Age (ref: 20 years)**						
18	1	−0.1072	0.3258	0.7422	1.792	0.707–4.541
19	1	−0.3238	0.2986	0.2782	1.443	0.603–3.456
21	1	0.0346	0.2617	0.8949	2.065	0.901–4.733
22–23	1	0.6896^*^	0.2456	0.0050	3.976	1.776–8.903
24–31	1	0.1871	0.2597	0.4712	2.406	1.032–5.610
32+	1	0.2105	0.2879	0.4646	2.463	1.003–6.048
**Race/ethnicity (ref: Caucasian)**						
African American	1	0.6777^*^	0.2355	0.0040	2.962	1.649–5.321
Hispanic	1	−0.2013	0.2261	0.3732	1.230	0.694–2.178
Other	1	−0.0683	0.3247	0.8334	1.405	0.600–3.287
**COVID-19 positive (ref: no)**						
Yes	1	0.1425	0.1218	0.2417	1.330	0.825–2.143
**Depression (ref: not depressed)**						
Depressed	1	0.3523^*^	0.1067	0.001	2.023	1.331–3.074
**Alcohol use (ref: never)**						
2–4 times/month	1	0.6997^*^	0.3329	0.0356	3.223	1.224–8.487
Monthly	1	−0.2290	0.2328	0.3253	1.273	0.746–2.172
**First-gen status (ref: yes)**						
No	1	0.0711	0.1130	0.5290	1.153	0.740–1.795
**Degree level (ref: undergrad)**						
Graduate	1	−0.2875	0.4021	0.4745	0.998	0.590–1.687
Other	1	0.7611	0.9913	0.4426	2.847	0.218–37.229
Postgraduate	1	−0.1885	0.6222	0.7619	1.101	0.269–4.507
**Residence (ref: on-campus)**						
Off-campus	1	−0.1911	0.1484	0.1977	0.682	0.381–1.221

^*^Statistically significant at the level of significance equal to 0.05.

### 3.3 Main results

Logistic regression analysis was conducted to examine the associations between demographic and behavioral factors and drug use (Table 1). The results indicated that female participants had lower odds of drug use compared to males [odds ratio (OR) = 0.814; 95% confidence interval (CI): 0.505–1.312], although this association did not reach statistical significance. In terms of racial/ethnic differences, African American students were more likely to report drug use compared to Caucasian students (OR = 2.962; 95% CI: 1.649–5.321) compared to white students. Additionally, students who reported experiencing depression had increased odds of drug use (OR = 2.023; 95% CI: 1.331–3.074). Alcohol consumption was also one of the significant predictors: individuals who consumed alcohol two to four times per month were more likely to use drugs compared to non-drinkers (OR = 3.223; 95% CI: 1.224–8.487). Regarding age, students aged 22 and 23 demonstrated higher odds of drug use relative to those aged 18 (OR = 3.98; 95% CI: 1.776–8.903) compared to 18-year-olds.

### 3.4 Other analysis

**Figure 1:**
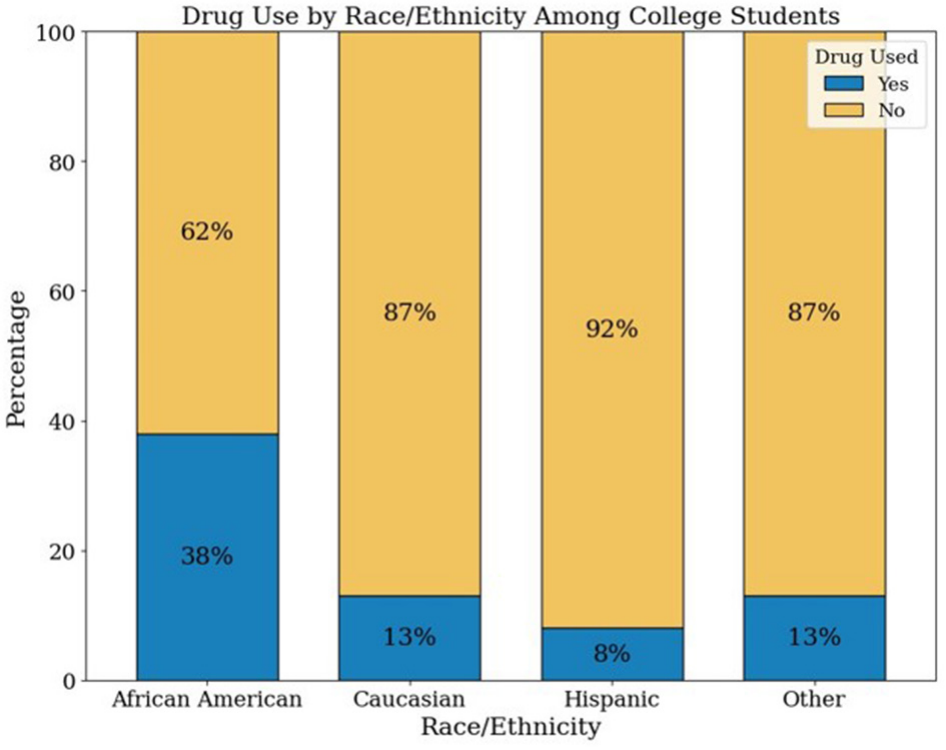
Percentage of students reporting drug use by race/ethnicity (*n* = 576).

**Figure 2:**
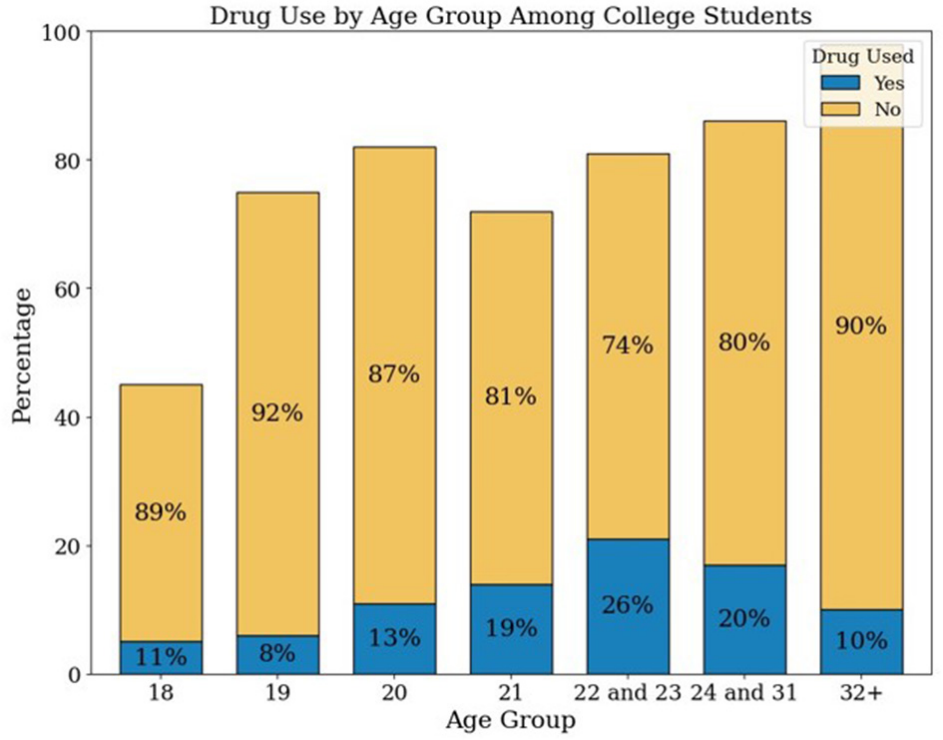
Percentage of drug use across age groups among college students (*n* = 576).

**Figure 3:**
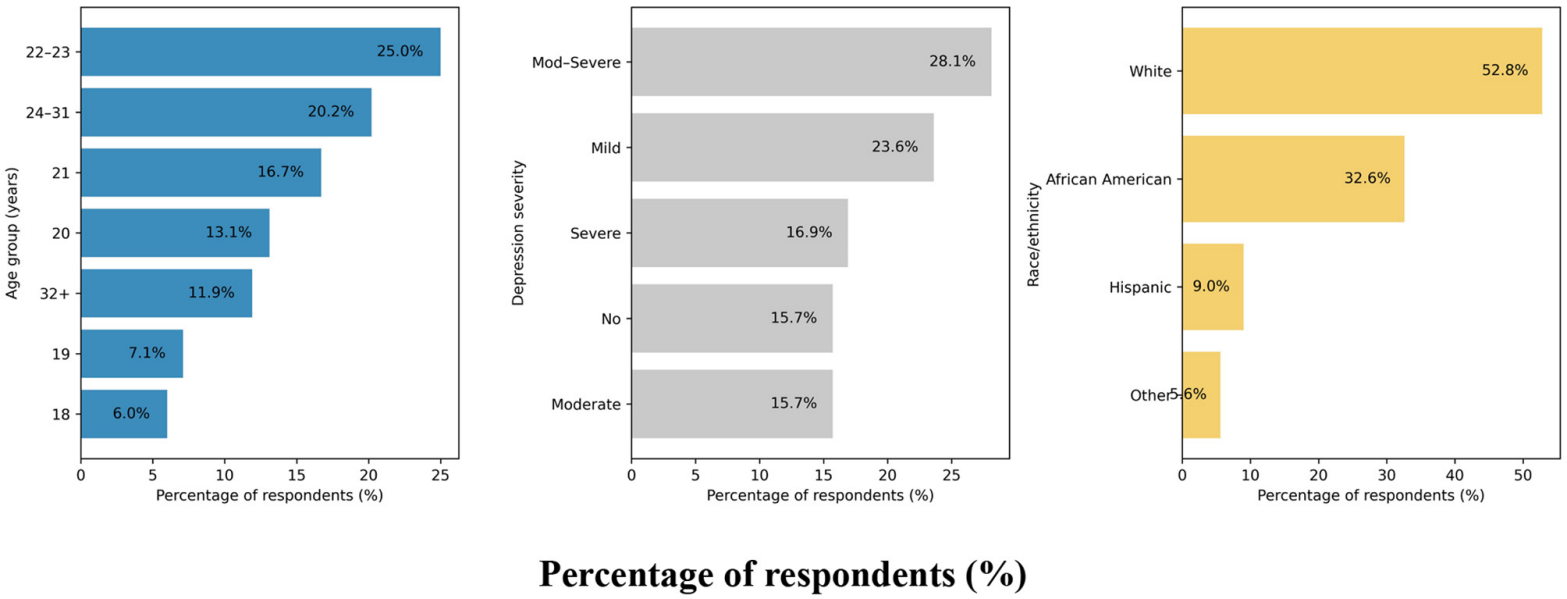
Proportional distribution of respondents by age group, depression severity, and race/ethnicity (*n* = 576).

**Figure 4:**
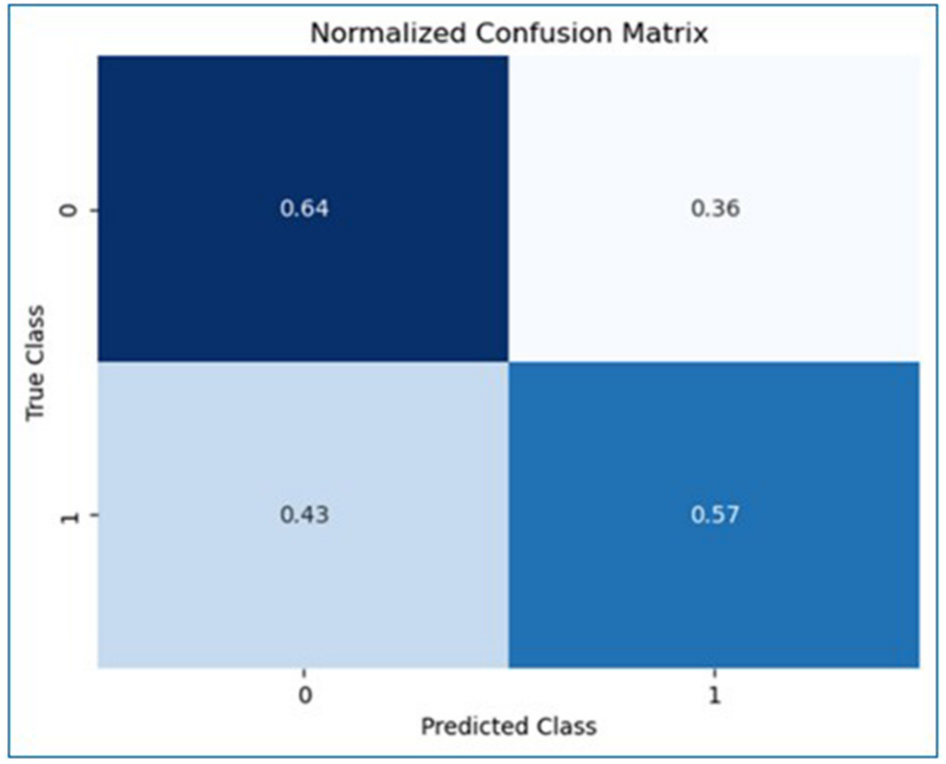
Normalized confusion matrix to evaluate the random forest model performance.

**Figure 5:**
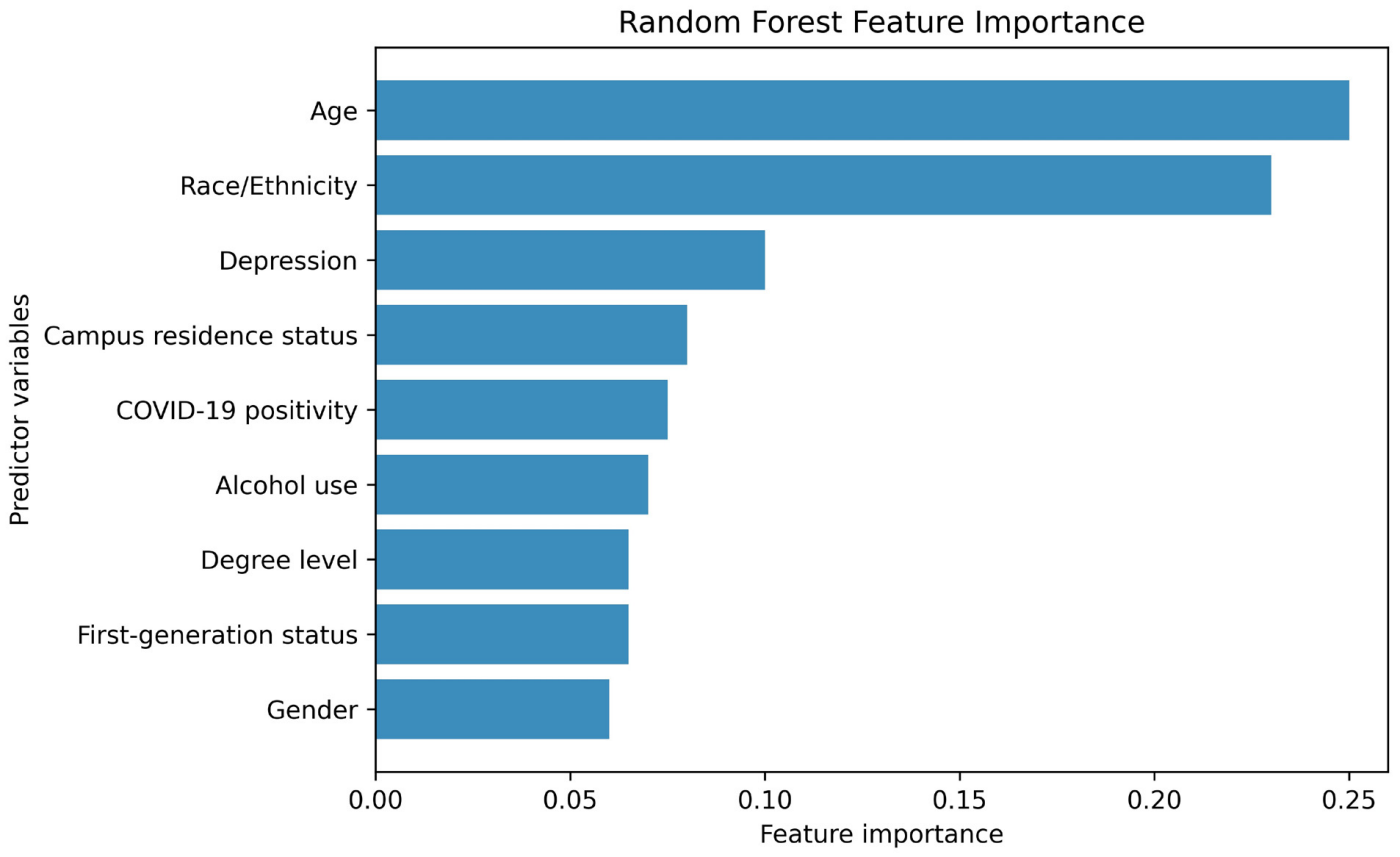
Feature importance derived from a Random Forest model based on mean decrease in impurity. Higher values indicate greater contribution to classification decisions.

Supplementary Table S2 presents the distribution of DAST-20 scores and corresponding intervention recommendations based on the ASAM Placement Criteria. Most participants (69.18%) fell within the “None” category; 27.44% were categorized as “Low” risk, and smaller proportions were classified as “Intermediate” (2.19%), “Substantial” (0.60%), or “Severe” (0.60%). Supplementary Table S3 stratifies participant characteristics by DAST-20 drug use severity levels. A Random Forest classifier was applied to the imputed DAST-20 data. SMOTE was used to balance the minority class. The dataset was split into training (80%) and testing (20%) subsets. Model performance was evaluated using accuracy, precision, recall, and F1 score. Figure 4 presents the normalized confusion matrix, showing 64% accuracy for non-risk and 57% for risk classification. Figure 5 ranks feature importance, with age as the top predictor, followed by race/ethnicity and depression symptoms. Gender had the lowest importance. Figures 1, 2 show drug use distribution by race/ethnicity and age, respectively, while Figure 3 illustrates the intersection of age, race/ethnicity, and depression severity. Supplementary Figure S1 shows the distribution of DAST-20 scores before and after oversampling.

Supplementary Table S1 presents the results of bivariate analyses examining the associations between drug use and various demographic, behavioral, and other factors (*n* = 576). Chi-square tests were employed to assess the independence between drug use status and categorical variables, including age group, race/ethnicity, gender, alcohol use, COVID-19 test result, first-generation college status, degree level, campus residency, and depression status. This statistical approach was selected due to its suitability for evaluating relationships between categorical variables and the outcome of interest (drug use).

Among the 576 participants who had complete data on mental health and drug use, 89 individuals (15.45%) reported using non-prescription drugs. Drug use prevalence was higher among men (20.81%) compared to women (13.11%). The highest rate of drug use was observed among students aged 22–23 years (25.93%). By race/ethnicity, African American students reported the highest prevalence of drug use (38.16%). Descriptive statistics indicated that the majority of respondents (71.53%) identified as women. Most participants were undergraduate students (71.48%), and nearly half (44.10%) identified as first-generation college students. Additionally, only 31.65% of respondents reported living on campus at the time of the survey.

Chi-square test results indicate statistically significant associations between drug use and several covariates among college students (Supplementary Table S1). Specifically, age, race/ethnicity, gender, alcohol use, and depression status were significantly associated with drug use patterns (*p* < 0.05), suggesting these variables may serve as important predictors in understanding substance use behaviors within this population. In contrast, no significant associations were found for COVID-19 positivity, first-generation college status, degree level, or campus residency.

Among students who reported drinking alcohol two to four times per month, 50% also reported drug use, compared to only 12.04% among non-drinkers. Additionally, 23.61% of participants tested positive for COVID-19, with 16.18% reporting drug use. Regarding mental health, 38.39% screened positive for depression. Among those with depressive symptoms, 24.11% reported drug use, compared to 10.67% among those without symptoms.

Figure 1 displays the proportion of students reporting drug use (yes vs. no) across four racial/ethnic groups: African American, Caucasian, Hispanic, and Other. African American students reported the highest prevalence of drug use (38%), followed by those identifying as Other (13%), Caucasian (13%), and Hispanic (8%).

Figure 2 illustrates the distribution of drug use (yes vs. no) across age groups among college students. The highest frequency of reported drug use was observed among students aged 22–23 years (*n* = 21), followed by those aged 24–31 years (*n* = 17) and 21 years (*n* = 14). In contrast, younger students aged 18 and 19 reported substantially lower frequencies of drug use, with only 5 and 6 individuals, respectively, indicating use. Notably, although the 32-year and older age group comprised the largest number of respondents (*n* = 98), only 10 individuals within this group reported drug use. Figure 3 illustrates the distribution of respondents by age, race/ethnicity, and severity of depression.

Supplementary Table S2 presents the distribution of DAST-20 scores among the sample (*n* = 503) and the corresponding intervention recommendations based on the American Society of Addiction Medicine (ASAM) Placement Criteria (70). The majority of participants (69.18%) fell within the “None” category; 27.44% were categorized as “Low” risk, for whom brief counseling (ASAM Level I) may be appropriate. A smaller proportion of participants were classified as “Intermediate” (2.19%), “Substantial” (0.60%), or “Severe” (0.60%), suggesting the need for outpatient or intensive treatment interventions, ranging from ASAM Levels I–IV.

Supplementary Table S3 presents participant characteristics stratified by DAST-20 drug use severity levels.

Figure 4 presents the normalized confusion matrix evaluating the model's classification performance for drug use risk (DAST-20). The model correctly identified 64% of participants with no reported drug use risk (DAST-20 = 0) and 57% of those with any level of risk (DAST-20 ≥ 1). However, 43% of at-risk participants were misclassified as not at risk, and 36% of non-risk participants were misclassified as at risk.

Figure 5 presents the ranked feature importance of variables in the Random Forest model used to classify DAST-20 outcomes. Age emerged as the most influential predictor, followed closely by race/ethnicity, highlighting the significant role of demographic factors in substance use risk. Depression symptoms were the third most important variable, consistent with prior research linking mental health and substance use. Other contributing predictors included campus residence, post-COVID status, alcohol use, degree level, first-generation college status, and gender. Notably, gender demonstrated the lowest feature importance, suggesting a relatively minor role in the model's predictive performance.

Supplementary Figure S1 demonstrates the distribution of binary DAST-20 scores before and after oversampling. Prior to oversampling, there was a clear class imbalance, with 348 participants categorized as *No Indication* and 155 as *At Risk*. Following oversampling, both categories were balanced to 348 participants each, ensuring equal representation and reducing potential bias in subsequent modeling.

## 4 Discussion

Substance use remains a significant public health issue in the United States, with national survey data consistently showing that usage rates are highest during the period of emerging adulthood (71–73). This study explored how demographic and psychosocial factors intersected with substance use behaviors among college students during the early stages of the COVID-19 pandemic, a time when the pandemic caused significant psychological stress worldwide, but had a particularly unique impact on college students (47, 54, 74). Specifically, we investigated which characteristics of college students were linked to higher substance use, assessed the overall prevalence of such behaviors, and examined their potential relationship with psychological wellbeing.

The rationale for this study is based on the idea that the start of the COVID-19 pandemic might have worsened existing vulnerabilities among college students, a group already seen as being at higher risk for mental health problems and substance use (47, 54, 55, 62, 74, 75). The key stressors documented in the literature during the pandemic were social isolation, academic disruptions, and economic uncertainty, all of which may have contributed to a heightened dependence on substances as a means of coping (47, 54, 55, 62, 74–77). By identifying key demographic and contextual factors associated with substance use, this study was conducted with the aim of pinpointing vulnerable student populations for future interventions. Such insights can support the development of more targeted strategies that address the unique challenges students face in balancing academic demands and basic needs during large-scale public health crises (5, 47, 74).

Substance use remains a common issue on U.S. college campuses (1–5, 78). For many young people, college marks a significant life transition, signifying the shift from adolescence to independence and adulthood. It is also a vulnerable time with heightened exposure to various illicit and prescribed substances (5, 47, 74, 79). In our study, college students aged 22–23 exhibited three times the odds of drug use compared to those aged 18. These findings highlight that substance use may vary by age during major outbreaks like COVID-19, emphasizing age-specific vulnerabilities (80–83). Before the pandemic, college campuses across the United States had witnessed a rise in student use of cannabis, stimulants, and other illicit substances (5).

According to the recent data, over half (55.7%) of American college students are current drinkers. Among current drinkers, approximately half (49.5%) report being drunk within the last 30 days. As for the co-consumption (alcohol and drugs), students who consumed alcohol two to four times per month were more likely to report drug use (50%) compared to non-drinkers (12.04%). This pattern reflects well-documented co-occurrence between alcohol and other substance use among college students. Data indicate that students initiating alcohol use earlier and those embedded in Greek life or who overestimate peer drinking are also more likely to engage in polysubstance use (1, 84). According to another recent national survey, nearly half of current drinkers report being drunk within the last 30 days, and one out of every 10 American college students reported consuming more than 10 drinks in a row at some point over the last 2 weeks (1, 19). The overlapping psychological and social factors, including sensation-seeking, peer influence, and impaired decision-making under the influence of alcohol, could potentially drive this association. Alcohol consumption can lower inhibitions and increase impulsivity, making individuals more likely to experiment with or escalate use of other substances (85–88). In our study, participants who reported consuming alcohol 2–4 times per month had threefold higher odds of substance abuse compared to those who reported no alcohol consumption.

In the present study, approximately 40% of students screened positive for depressive symptoms. The odds of drug use in this subgroup were nearly twice that observed among students without depressive symptoms. This finding aligns with a substantial body of research indicating that depression significantly increases vulnerability to substance use, often as a form of self-medication to manage emotional distress (89–91). The self-medication hypothesis suggests that individuals with mood and anxiety disorders may turn to substances such as alcohol or drugs to alleviate psychological discomfort, which can, over time, lead to the development of substance use disorders (SUDs) (89). However, according to the literature, the relationship between mental health and substance use may be bidirectional. While depression may increase the risk of substance use, chronic substance use can also exacerbate or even trigger depressive symptoms, creating a reinforcing cycle of psychological distress and maladaptive coping (76, 92, 93). This feedback loop underscores the importance of integrated mental health and substance use interventions, particularly in college populations where both issues are prevalent. During periods of heightened stress, such as public health crises, students may face compounded pressures related to academic performance, financial insecurity, and social isolation, all of which can exacerbate both depressive symptoms and substance use behaviors (76, 94). Targeted prevention and support strategies that consider these intersecting factors are essential for promoting student wellbeing and reducing long-term health risks.

Although prior research has consistently reported racial and ethnic variations in drug use prevalence among college students, typically showing higher usage rates among white students (95), our findings diverge from this trend. In our sample, African American students reported the highest prevalence of drug use, followed by Hispanic and other racial/ethnic groups. However, this pattern aligns with emerging research suggesting that African American youth may be at elevated risk for polydrug use, particularly in environments where substances such as alcohol, opioids, and stimulants are readily accessible. A recent mixed-methods study found that African Americans who misuse opioids frequently co-use substances like alcohol, cocaine, and methamphetamine, often to enhance intoxication or mitigate side effects, behaviors that may persist into the college environment (96). These disparities are further exacerbated by cumulative stressors—such as structural inequities and adverse childhood experiences or adolescent stressful life events (97, 98) that disproportionately impact students of color. The convergence of academic pressures, limited access to culturally responsive mental health services, and broader systemic inequalities may exacerbate substance use behaviors among African American and Hispanic students (99). As such, these findings underscore the importance of tailored prevention and intervention strategies that address both individual risk factors and the broader social determinants of health.

The finding that men reported higher rates of non-prescription drug use than women is consistent with a well-established body of research on gender differences in risk-taking and substance use (100–104). However, we were unable to demonstrate this association in our study. A meta-analysis of 150 studies demonstrated that males consistently engage in higher levels of risk-taking behavior across various domains, including smoking and illicit drug use, with significant effect sizes observed across age groups (105). More recent analyses continue to show that men are more likely to meet criteria for SUDs, particularly for substances such as cocaine and cannabis, despite a narrowing gender gap in overall use (106). These patterns are shaped by a combination of biological and sociocultural influences. Biologically, differences in brain structure, hormonal regulation, and metabolic processing may contribute to men's increased susceptibility to initiating and maintaining substance use (107). Socio-culturally, norms surrounding masculinity and sensation-seeking behaviors reinforce risk-taking tendencies among men, further elevating their likelihood of engaging in substance use (108, 109). Literature also highlights gender differences in coping with perceived stress by college students (110) that potentially influence substance use behaviors.

### 4.1 Strengths

A notable strength of this study lies in the predictive performance of the logistic regression model. With a concordance rate of 80% and a c-statistic of 0.801, our model demonstrated strong discriminatory power in distinguishing between college students who reported drug use and those who did not (111). These metrics suggest that the model identified key predictors of drug use, supporting the robustness of the analytical approach and enhancing confidence in the validity of the findings. Another strength of this study is the reduced likelihood of recall bias (112). Data were collected during the height of the COVID-19 pandemic, a period when students were actively experiencing its effects. As a result, their responses likely reflect real-time perceptions, emotions, and behaviors, enhancing the authenticity and accuracy of self-reported data. As such, the immediacy of the stressors likely encouraged more genuine disclosure of mental health challenges, substance use behaviors, and related experiences. The use of a large and geographically diverse sample of the student body enhances the generalizability of the findings across varied institutional and regional contexts. Additionally, the application of validated mental health and substance use and abuse instruments, such as the PHQ-9 and DAST-20, supports the reliability and validity of the measured outcomes. By focusing on college students, a population uniquely vulnerable to pandemic-related stressors, the study addresses an often-underrepresented group in substance abuse research. Despite its cross-sectional design, the use of rigorous statistical analyses, including multivariable logistic regression, further strengthens the study's ability to identify meaningful associations while adjusting for potential confounders.

### 4.2 Limitations

Several limitations should be acknowledged when interpreting the findings of this study. First, the cross-sectional design precludes causal inference, limiting the ability to determine the temporal direction of observed associations between pandemic-related stressors and substance abuse behaviors (113). Second, reliance on self-reported data introduces the potential for response bias, including social desirability (114) and recall bias (112), which may have impacted the measures of association, as students might have been reluctant to fully disclose sensitive information related to mental health challenges or substance use behaviors. Hence, the reported odds could be an underestimation of the actual associations. Additionally, the absence of pre-pandemic baseline mental health data restricts the ability to quantify changes directly attributable to the COVID-19 crisis. The use of non-probability, convenience sampling may also have led to selection bias (115), as students experiencing higher levels of psychological distress may have been more likely to participate. Although multivariable analyses were conducted, the possibility of unmeasured confounding remains. Some factors, such as child abuse, personality disorders, shifts in family environments, dependency issues, and suicidal thoughts, are linked to substance abuse in adolescents and young adults (116). We could not study these factors in the explored associations. Finally, while the sample was geographically diverse, the findings of our study may not be generalizable to public universities in different states, as well as historically Black colleges or universities, or Hispanic Service Institutions. The characteristics of our sample, like those of many others describing college students during this time, showed an overrepresentation of white participants and females, but this is consistent with the demographic breakdown of the university where the study was conducted (74, 117, 118). One key limitation of this study is the low response rate to sensitive items, particularly those related to drug use. This could potentially reduce statistical power and introduce nonresponse bias, as individuals engaging in higher-risk behaviors may be less likely to disclose such information (119). To mitigate this limitation, we applied the *Amelia* multiple imputation method (69).

Stratified analyses (Supplementary Table S3) revealed contextual factors associated with substance abuse risk, including gender, race/ethnicity, alcohol use, degree level, and depression status. While these associations are consistent with prior research on social determinants of substance use (115, 120), the sparse data in our study limits confidence in these findings. Low response rates to sensitive questions, such as drug use, may have introduced selection bias, with individuals experiencing higher distress potentially more likely to participate or disclose such behaviors (121). Future studies with more robust sampling and higher response rates are needed to confirm these patterns and strengthen the evidence base.

Substance misuse among college students is associated with a range of serious consequences that extend beyond academic impacts. These include declining academic performance, increased engagement in risky behaviors, and adverse physical and mental health outcomes such as addiction and suicidal ideation. Additionally, substance use is associated with a higher risk of sexual assault, both perpetration and victimization, and may contribute to long-term challenges such as unemployment after graduation (5, 37, 122). Additionally, students using substances may face social consequences, including damaged relationships and increased isolation. Substance use initiated during college often persists into young adulthood and beyond, underscoring this developmental stage as a critical window for the formation of long-term patterns of drug use.

Collectively, these findings emphasize the importance of systematic screening and early intervention within college health systems. Tailored strategies that address mental health, substance use, and underlying sociodemographic disparities are warranted to mitigate long-term consequences and promote student wellbeing. Our findings highlight the importance of early identification and intervention among at-risk students to mitigate potential academic, health, and safety consequences associated with sustained substance use (122).

## 5 Conclusions

College students are a particularly vulnerable group when it comes to substance use, especially during times of increased stress like public health crises. This study supports the growing evidence that mental health issues, particularly depression and distress, are closely connected to higher substance use among students. These behaviors may serve as coping strategies in response to academic pressures, social isolation, financial strain, and other stressors that uniquely impact young adults in higher education.

Our findings emphasize the importance of addressing substance use within a broader framework that includes students' mental health, demographic backgrounds, and changes in their financial and basic needs. By identifying key factors related to substance use, such as depressive symptoms as well as age, gender or racial differences, institutions can better customize prevention and intervention strategies to suit the diverse needs of their student populations. Integrated approaches that combine mental health support with substance use education and services are crucial for enhancing student wellbeing and academic success.

## Data Availability

The datasets presented in this study can be found in online repositories. The names of the repository/repositories and accession number(s) can be found at: 10.17632/bvxm22n8rx.1.
