# Supplementary material for: Demographic and other correlates of non-prescription drug use among college students during the COVID-19 pandemic
Source: Front Public Health. 2026 Feb 4;13:1695969. doi: 10.3389/fpubh.2025.1695969 (PMC12913380; doi:10.3389/fpubh.2025.1695969)
Supplement: Supplementary file 1 [file Table_1.pdf]

Table S1: Associations Between Participant Characteristics and Drug Use Status as Assessed by DAST-20 (n = 576)

**Variables**	**Participants (n, %)** (N=576)	**Did not use drugs** (N=487)	**Used drugs** (N=89)	**Chi-Square Test** P-value
Gender				
Male	149 (25.87%)	118 (79.19%)	31 (20.81%)	0.0398*
Female	412 (71.53%)	358 (86.89%)	54 (13.11%)	
Others	15 (2.60%)	11 (73.33%)	4 (26.67%)	
Age				
18	45 (8.35%)	40 (88.89%)	5 (11.11%)	0.0206*
19	75 (13.91%)	69 (92%)	6 (8%)	
20	82 (15.21%)	71 (86.59%)	11 (13.41%)	
21	72 (13.36%)	58 (80.56%)	14 (19.44%)	
22 - 23	81 (15.03%)	60 (74.07%)	21 (25.93%)	
24 - 31	86 (15.96%)	69 (80.23%)	17 (19.77%)	
32+	98 (18.18%)	88 (89.80%)	10 (10.20%)	
Race and Ethnicity				
Caucasian	356 (61.81%)	309 (86.80%)	47 (13.20%)	0.0000*
African American	76 (13.19%)	47 (61.84%)	29 (38.16%)	
Hispanic	105 (18.23%)	97 (92.38%)	8 (7.62%)	
Others	39 (6.77%)	34 (87.18%)	5 (12.82%)	
First Generation Students				
Yes	254 (44.10%)	222 (87.40%)	32 (12.60%)	0.1136
No	321 (55.73%)	264 (82.24%)	57 (17.76%)	
Missing	1 (0.17%)			

Degree Level				
Undergraduate	411 (71.48%)	347 (60.24%)	64 (11.11%)	0.2206
Graduate	146 (25.39%)	124 (84.93%)	22 (15.07%)	
Postgraduate	14 (2.53%)	13 (92.86%)	1 (7.14%)	
Others	4 (0.70%)	2 (50%)	2 (50%)	
Missing	1 (0.17%)			
Campus Residence				
On Campus	182 (31.65%)	152 (83.52%)	30 (16.48%)	0.7417
Off Campus	393 (68.35%)	334 (84.99%)	59 (15.01%)	
Missing	1 (0.17%)			
Alcohol Use				
2-4 times/Month	18 (3.12%)	9 (50%)	9 (50%)	0.0000*
Month	91 (15.80%)	66 (72.53%)	25 (27.47%)	
Never	457 (79.34%)	402 (87.96%)	55 (12.04%)	
Missing	10 (1.74%)			
Depression Status				
Yes	224 (38.89%)	170 (75.89%)	54 (24.11%)	0.0000*
No	328 (56.94%)	293 (89.33%)	35 (10.67%)	
Missing	24 (4.16%)			
Anxiety Status				
Yes	226 (39.24%)	168 (74.34%)	58 (25.66%)	0.0000*
No	326 (56.60%)	295 (90.49%)	31 (9.51%)	
Missing	24 (4.16%)			
Positive Covid-19 test				
Yes	136 (23.61%)	114 (83.82%)	22 (16.18%)	0.8950
No	440 (76.39%)	373 (84.77%)	67 (15.23%)	
