# Supplementary material for: Demographic and other correlates of non-prescription drug use among college students during the COVID-19 pandemic
Source: Front Public Health. 2026 Feb 4;13:1695969. doi: 10.3389/fpubh.2025.1695969 (PMC12913380; doi:10.3389/fpubh.2025.1695969)
Supplement: Supplementary file 2 [file Table_2.pdf]

Table S2: Drug Abuse Screening Test-20 (DAST-20) Severity Categories, Recommended Clinical Actions, and ASAM Levels of Care (n = 503)

DAST	Frequency	Percent	Action	**ASAM:** American Society of Addiction Medicine Placement Criteria
**None**	348	69.18	Monitor	
**Low**	138	27.44	Brief Counseling	Level I
**Intermediate**	11	2.19	Outpatient (Intensive)	Level I or II
**Substantial**	3	0.60	Intensive	Level II or III
**Severe**	3	0.60	Intensive	Level III and IV
