# Supplementary material for: Demographic and other correlates of non-prescription drug use among college students during the COVID-19 pandemic
Source: Front Public Health. 2026 Feb 4;13:1695969. doi: 10.3389/fpubh.2025.1695969 (PMC12913380; doi:10.3389/fpubh.2025.1695969)
Supplement: Supplementary file 3 [file Table_3.pdf]

Table S3: Associations Between Participant Characteristics and DAST-20 Substance Use Severity Categories (n = 503).

**Variables**	**Participants (N=503)**	**None (N=348)**	**Low (N=138)**	**Intermediate (N=11)**	**Substantial (N=3)**	**Severe (N=3)**	**P-Value**
Gender							0.0461*
Male	130	86 (66.15%)	37 (28.46%)	5 (3.85%)	1 (0.77%)	1 (0.77%)	
Female	359	255 (71.03%)	95 (26.46%)	6 (1.67%)	1 (0.28%)	2 (0.56%)	
Others	14	7 (50%)	6 (42.86%)	0 (0%)	1 (7.14%)	0 (0%)	
Age							0.0715
18	43	31 (72.09%)	11 (25.58%)	0 (0%)	1 (2.33%)	0 (0%)	
19	73	55 (75.34%)	15 (20.55%)	1 (1.37%)	0 (0%)	2 (2.74%)	
20	70	56 (80%)	12 (17.14%)	2 (2.86%)	0 (0%)	0 (0%)	
21	67	46 (68.66%)	19 (28.36%)	2 (2.86%)	0 (0%)	0 (0%)	

22 - 23	72	38 (52.78)	30 (41.67%)	4 (5.56%)	0 (0%)	0 (0%)	
24 - 31	72	48 (66.67%)	21 (29.17%)	1 (1.39%)	1 (1.39%)	1 (1.39%)	
32+	78	58 (74.36%)	19 (24.36%	0 (0%)	1 (1.28%)	0 (0%)	
Missing	28						
Race and Ethnicity							<0.0001*
Caucasian	312	231 (74.04%)	75 (24.04%)	5 (1.60%)	0 (0%)	1 (0.32%)	
African American	67	30 (44.78%)	31 (46.27%)	3 (4.48%)	1 (1.49%)	2 (2.99%)	
Hispanic	93	66 (70.97%)	25 (26.88%)	2 (2.15%)	0 (0%)	0 (0%)	
Others	31	21 (67.74%)	7 (22.58%)	1 (3.23%)	2 (6.45%)	0 (0%)	
First Generation Students							0.6659
Yes	217	154	55	4	2	2	

		(70.97%)	(25.35%)	(1.84%)	(0.92%)	(0.92%)	
No	285	193<br>(67.72%)	83<br>(29.12%)	7<br>(2.46%)	1<br>(0.35%)	1<br>(0.35%)	
Missing	1						
Degree Level							0.0426*
Undergraduate	364	253<br>(69.51%	98<br>(26.92%)	10<br>(2.75%)	2<br>(0.55%)	1<br>(0.27%)	
Graduate	124	85<br>(68.55%)	36<br>(29.03%)	1<br>(0.81%)	0<br>(0%)	2<br>(1.61%)	
Postgraduate	11	8<br>(72.73%)	2<br>(18.18%)	0<br>(0%)	1<br>(9.09%)	0<br>(0%)	
Others	3	1<br>(33.33%)	2<br>(33.33%)	0<br>(0%)	0<br>(0%)	0<br>(0%)	
Missing	1						
Campus Residence							0.7730
On Campus	170	114<br>(67.06%)	49<br>(28.82%)	4<br>(2.35%)	1<br>(0.59%)	2<br>(1.18%)	
Off Campus	332	233<br>(70.18%)	89<br>(26.81%)	7<br>(2.11%)	2<br>(0.60%)	1<br>(0.30%)	

Missing	1							
Alcohol Use								0.0236*
2-4 times/Month	17	7<br>(41.18%)	8<br>(47.06%)	2<br>(0.51%)	0<br>(0%)	1<br>(5.88%)		
Month	86	54<br>(62.79%)	28<br>(32.56%)	2<br>(0.51%)	1<br>(1.16%)	0<br>(0%)		
Never	394	283<br>(71.83%)	100<br>(25.38%)	7<br>(1.78%)	2<br>(0.51%)	2<br>(0.51%)		
Missing	6							
Depression Status								0.0005*
Yes	207	120<br>(57.97%)	78<br>(37.68%)	7<br>(3.38%)	1<br>(0.48%)	1<br>(0.48%)		
No	281	214<br>(76.16%)	59<br>(21%)	4<br>(1.42%)	2<br>(0.71%)	2<br>(0.71%)		
Missing	15							
Anxiety Status								<0.0001*
Yes	187	105<br>(56.15%)	71<br>(37.97%)	8<br>(4.28%)	2<br>(1.07%)	1<br>(0.53%)		

No	301	229 (76.08%)	66 (21.93%)	3 (1%)	1 (0.33%)	2 (0.66%)	
Missing	15						
Positive Covid-19 test							0.2325
Yes	115	76 (66.09%)	33 (28.70%)	4 (3.48%)	0 (0%)	2 (1.74%)	
No	388	272 (70.10%)	105 (27.06%)	7 (1.80%)	3 (0.77%)	1 (0.26%)	

*Statistically significant at the level of significance equal to 0.05
