# Supplementary figures and images for: Demographic and other correlates of non-prescription drug use among college students during the COVID-19 pandemic
Source: Front Public Health. 2026 Feb 4;13:1695969. doi: 10.3389/fpubh.2025.1695969 (PMC12913380; doi:10.3389/fpubh.2025.1695969)

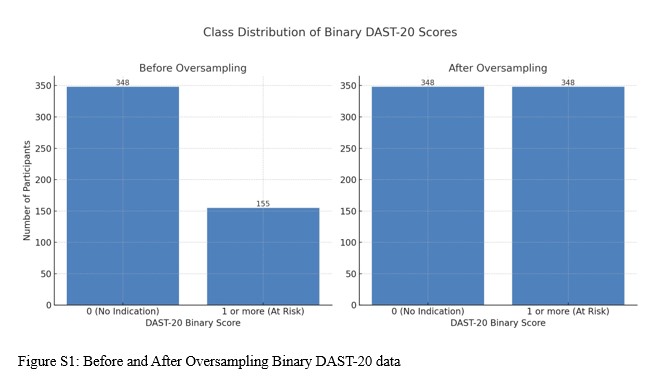

Supplement: Supplementary file 4 [file Image_1.jpeg]
